# Hydrothermal preparation of high purity TiO_2_ from industrial metatitanic acid by response surface methodology

**DOI:** 10.1038/s41598-022-24661-0

**Published:** 2022-11-23

**Authors:** Congxue Tian

**Affiliations:** 1 School of Vanadium and Titanium, Panzhihua University, Panzhihua, 617000 Sichuan People’s Republic of China; 2 Vanadium and Titanium Resource Comprehensive Utilization Key Laboratory of Sichuan Province, Panzhihua University, Panzhihua, 617000 Sichuan People’s Republic of China

**Keywords:** Chemistry, Materials science

## Abstract

The response surface methodology of Box Behnken design was used to investigate the effects of hydrothermal conditions on the high purity TiO_2_ preparation from industrial metatitanic acid. The method had a good fitting result in the prediction model, and the effects could be calculated from a second-order polynomial equation. The hydrothermal conditions greatly affected the structure and purity for the metatitanic acid and rutile TiO_2_, influenced the process of nucleation and crystallization, grain growth, polymerization, agglomeration and aggregation, further improved the particle size distribution, structure and surface adsorption capacity of metatitanic acid, reduced the adsorption of impurity ions, and finally improved the purity of TiO_2_. The variables such as hydrothermal temperature, slurry concentration and hydrothermal time had synergistic effects, and the effects of hydrothermal time were larger than the other two. The verification experiments confirmed that the predicted values could be achieved at 99.99% under the optimal hydrothermal conditions.

## Introduction

High purity titanium dioxide (TiO_2_) with TiO_2_ content higher than 99.8*%*, has been known for its unique excellent structure and performances, excellent semiconductor characteristics and electronic material properties, widely used in many functional material fields, such as electronic materials, electronic ceramics, capacitors, titanium dioxide based catalyst carriers, energy conversion devices, photoelectric conversion devices, carbon dioxide conversion catalysts, titanium alloys, titanium dioxide based functional materials, and so on, and when used for precision sensors and aerospace coatings, there is a higher requirement for the purity of TiO_2_^1–11^. Researchers had investigated the synthesis methods and conditions for the high purity TiO_2_ preparation, and there were four methods, mainly including titanium alkoxide hydrolysis, direct hydrolysis of titanium tetrachloride, the chloride process and the sulfate process, which had their own advantages and disadvantages^12–20^, as described in the reference^20^. The sulfate process was famous for its simple and practical technical approach, cheap and easily available mineral resources, and the process was simple and easy to control, while its disadvantage was that impurities are easily introduced in the preparation process, resulting in reducing the purity of TiO_2_. The sulfate process was the most promising preparation method for industrial applications. Metatitanic acid (H_2_TiO_3_, *MA*) was prepared from the hydrolysis process via the sulfate process at higher sulfuric acidity, higher impurity concentration (such as Fe^2+^, Mn^2+^, Cr^3+^, Al^3+^, et al.) by using industrial TiOSO_4_ solution as raw material. While *MA* had large specific surface area and colloidal properties, which could absorb a large amount of impurities, eventually reduce the purity of TiO_2_. Comparing with traditional sulfate process, using un-concentrated TiOSO_4_ solution instead of the concentrated could obtain narrower particle size distribution *MA* and good quality TiO_2_ pigments, which had the advantages of shortening the process flow, saving the energy consumption and reducing the production cost^21^. Hydrothermal method could improve the crystallinity of the matrix, reduce its specific surface area and colloidal properties, minimize the adsorption ability of *MA* to impurities and improve the purity of TiO_2_. Hydrothermal reaction process mainly included crystal growth, transformation and phase equilibrium, widely used in many fields of materials preparation. Hydrothermal treatment had been used to prepare porous TiO_2_ and nanometer materials with good performances and applications^22–24^. It would provide a simple way to control its particle size distribution, composition, structures and surface properties of *MA* by regulating and controlling the hydrothermal conditions. The composition and structure of high purity TiO_2_ prepared by hydrothermal method was strongly dependent on various complex effects preparation conditions, it would be necessary to develop experimental design to investigate and optimize these conditions.

Response surface methodology (*RSM*) was a statistical method to predict the response value by analyzing the regression equation and optimizing the process parameters, and was also used to check the influences of the selected parameters on the experimental results. *RSM* could reflect the interactive influences of different factors on the test results and make up for the influence of only single factor on the test results in the ordinary orthogonal optimization method. *RSM* had been widely used to solve many optimizations of photo-catalysis processes^25,26^. Besides, the modeling was an effective tool to determine the interaction of multiple variables and expand the experimental scale^27^. Using *RSM* to carry out statistical design of experiments was a useful way to conduct experiments, could have a basic idea about the range of variables, and the Box-Behnken design (*BBD*) was one of the more efficient tools^28^. The *BBD* was a response surface design type, which did not include embedded factor or partial factor design, which could investigate the interactions between factors and obtain optimal results under a small number of experiments.

As hydrothermal treatment could significantly reduce crystallinity and impurity adsorption capacity of *MA*, it was interesting to investigate the effects of hydrothermal conditions (such as slurry concentration, hydrothermal temperature and hydrothermal time) on the structure and purity of *MA* and TiO_2_. It would provide a theoretical basis for the high purity TiO_2_ preparation from the cheap and readily available industrial raw materials by using the *RSM* of Box-Behnken design method to investigate the hydrothermal conditions.

## Experimental

### Hydrothermal treatment

Hydrothermal treatment was used to prepare high purity TiO_2_ by using the hydrated metatitanic acid as raw material, which was obtained from the short sulfate process by hydrolysis of un-concentrated TiOSO_4_ solution as described in reference^29^. After hydrolysis, the obtained slurry was cooled to 70 °C, then filtered and washed at 65 °C with pure water, with 1:1 (volume ratio) as to the TiOSO_4_ solution, and obtained the hydrated metatitanic acid, and it was mainly composed of H_2_O and TiO_2_, the remaining impurities content was of about 4.17*%*, mainly composed the elements of S, Fe, Si, Al, Nb, Zn, V and so forth. Then the hydrated metatitanic acid was hydrothermal treated, filtered, washed and calcined. After determining the TiO_2_ mass content for the filter cake, the metatitanic acid cake was beaten to suspended slurry with deionized water to a certain mass concentration. Then the slurry was put into the hydrothermal reactor and sealed, with the filling degree of 85*%*. The hydrothermal reactor was placed into the oven at different hydrothermal temperature and for different hydrothermal time. After hydrothermal treatment, the reacting slurry was cooled to 70 °C, filtered and washed with the deionized water at 65 °C, with the volume ratio as to the slurry of 5:1, then obtained the hydrothermal treated metatitanic acid. The samples were calcined at air atmosphere in a muffle furnace, the calcining conditions were conducted as the following: at first from room temperature to 420 °C in 30 min and holding for 60 min, then from 420 to 850 °C in 90 min and holding for 150 min. The samples were cooled and grinded by a three head grinder, finally obtained the high purity product.

### Characterization

The inductively coupled plasma optical emission spectrometry (*ICP-OES*, iCAP 6300, Thermo Fisher, USA) was used to determine the content of all impurities, and the purity value of TiO_2_ were obtained by subtracting the content of all impurities from 100%. An X-ray diffractometer (X’ Pert3 Powder, PANalytical) was used to determine the crystal structures of the metatitanic acid and TiO_2_, and the crystal size for metatitanic acid and TiO_2_ were calculated according to the *Scherrer* equation. The particle size distribution (*PSD*) data of TiO_2_ were obtained from a particle size analyzer (Mastersizer 2000, Malvern). The surface and pore size distribution analyzer instrument (3H-2000PS1, Beishide, China) was used to determine the specific surface area (*S*_*BET*_) of metatitanic acid. The morphologies of the high purity TiO_2_ samples were observed by the field emission scanning electron microscopy (Sigma 500/VP, Zeiss, Germany).

## Results and discussions

### Experimental results and discussions

To investigate the optimum hydrothermal conditions for preparation of high purity TiO_2_, the experimental design as a function of the selected main factors had to be determined. The conditions were optimized by applying the *BBD* method and response surface methodology (*RSM*), including 17 experiments. Taking the purity of TiO_2_ (*Y*) as the response value, and the three main factors that significantly affected the purity of TiO_2_ including the slurry concentration (*X*_*1*_), hydrothermal temperature (*X*_*2*_) and hydrothermal time (*X*_*3*_) were treated as the investigation factors, the Box–Behnken test factors and levels were shown in Table 1. Minor changes in experimental operation would bring about changes in the purity of titanium dioxide, in order to ensure the accuracy of the experiments and the influence of the experimental error on the variance analysis of *RSM*, 17 experiments were set up. All the experimental runs of *BBD* were performed in accordance with Table 2, the test results and predicted values were shown in Table 2.

**Table 1:** Factors and levels of *BBD* experiments for hydrothermal conditions optimization for high purity TiO_2_.

Factors	− 1	0	1
Slurry concentration (g/L), *X*_1_	140	160	180
Hydrothermal temperature (*°*C), *X*_2_	120	140	160
Hydrothermal time (h), *X*_3_	4	6	8

**Table 2:** Experimental design matrix, experimental results and predicted purity of TiO_2_.

No	*X*_1_ (g/L)	*X*_2_ (°C)	*X*_3_ (h)	*Y* Measured (%)	*Y* Predicted (%)
1	140	120	6	99.56	99.57
2	180	120	6	99.34	99.34
3	140	160	6	99.78	99.79
4	180	160	6	99.62	99.61
5	140	140	4	98.93	98.91
6	180	140	4	98.84	98.84
7	140	140	8	99.81	99.81
8	180	140	8	99.45	99.47
9	160	120	4	98.71	98.72
10	160	160	4	99.09	99.10
11	160	120	8	99.64	99.63
12	160	160	8	99.73	99.72
13	160	140	6	99.99	99.96
14	160	140	6	99.96	99.96
15	160	140	6	99.91	99.96
16	160	140	6	99.97	99.96
17	160	140	6	99.95	99.96

**Figure 1:**
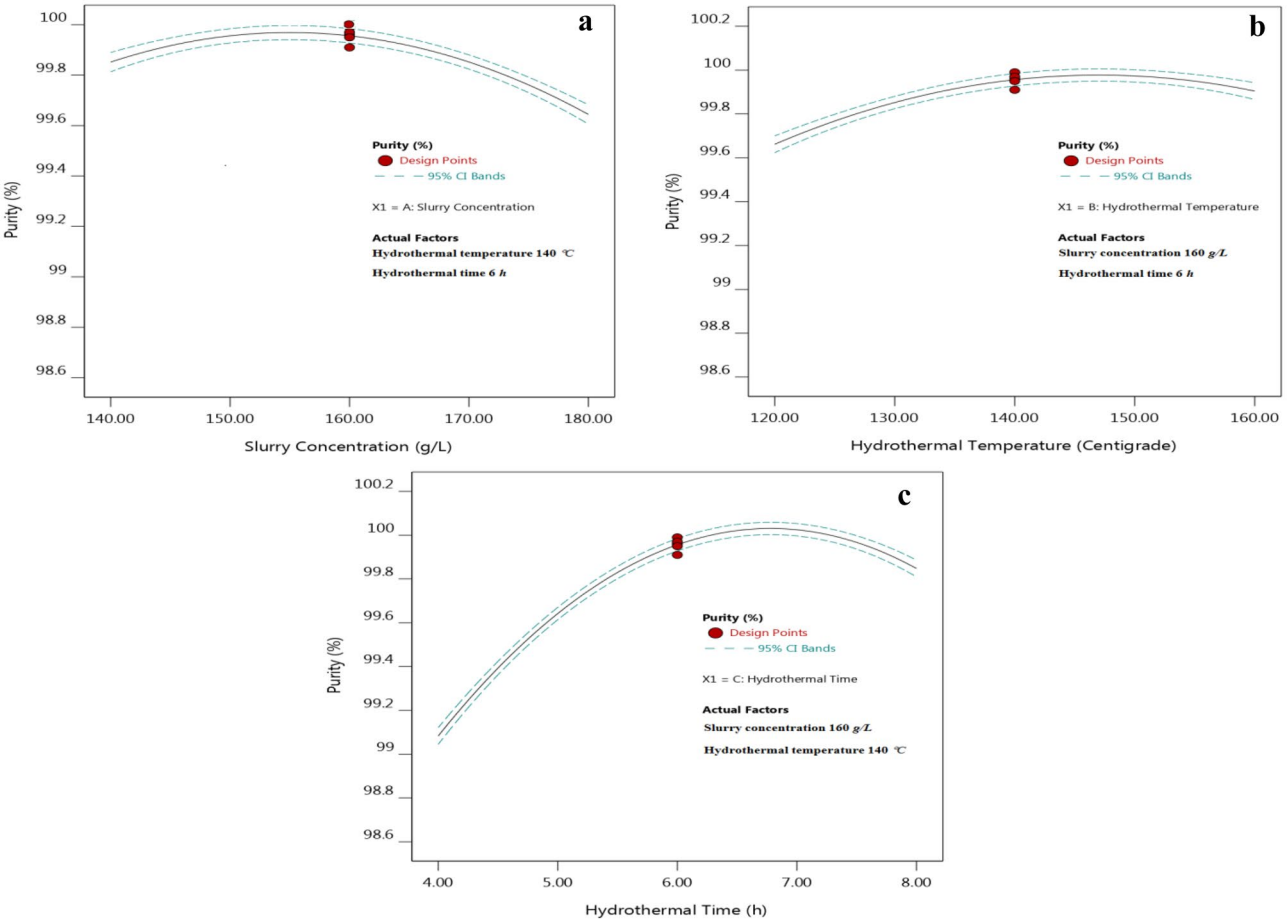
Effects of hydrothermal conditions on the purity of TiO_2_ (**a**) Hydrothermal temperature 140 °C, Hydrothermal time 6 h; (**b**) Slurry concentration 160 g/L, Hydrothermal time 6 h; (**c**) Slurry concentration 160 g/L, Hydrothermal temperature 140 °C*.*

In order to investigate the effects of single factor on the purity of TiO_2_, the other two factors could be fixed at the optimal value. Under the fixed hydrothermal temperature of 140 °C and hydrothermal time of 6 h, with the increasing of slurry concentration, the purity first increased and then decreased, and the results showed that there were multiple interactions between slurry concentration and other two factors, as showed in Fig. 1a. The slurry concentration was similar to the reactant concentration, which would affect the crystallization of TiO_2_, the diffusion of crystal structured TiO^2+^ ion, grain growth and aggregation, then influence the microstructure and impurities adsorption of metatitanic acid, and ultimately influence the structure and purity of TiO_2_. Suitable slurry concentration was favorable to obtain hydrothermal metatitanic acid with smaller crystal size about several nanometers, and it had extremely high surface energy as mentioned in the reference^30^, which was easier to form larger primary agglomerate particles, then to form smaller secondary aggregate particles, and the secondary aggregate particle corresponded to the average particle size of metatitanic acid, leading to reducing the impurity adsorption and beneficial to the preparation of high purity TiO_2_.

Under the fixed 160 g/L slurry concentration and 6 h hydrothermal time, with the increasing of hydrothermal temperature, the purity of TiO_2_ first increased and then decreased, and the results showed that there were interactions between hydrothermal temperature and the other two factors, as showed in Fig. 1b. Hydrothermal temperature had great effects on the crystallization activation energy, solubility of the precursors and super-saturation of the aggregates, eventually influenced the specific surface area, crystal structure and particle size distribution of the hydrothermal treated metatitanic acid. Increasing hydrothermal temperature could provide more energy, help to overcome energy barrier for phase transformation, strengthen the mass transport, and enhance the collision, combination, growth and aggregation of TiO^2+^ ions for the growth and crystal formation on the surface of anatase TiO_2_ crystals. All these effects promoted the crystal growth and particles aggregation for the hydrothermal products. Suitable hydrothermal temperature was favorable to obtain appropriate crystal size for metatitanic acid, while appropriate size crystals were more conducive to form larger primary agglomerate particles, then to form smaller secondary aggregate particles, consisted with the average particle size of metatitanic acid^30^. However, when the hydrothermal temperature was too low or too high, it was easy to precipitate and form some finer crystals and particles, which would make the particle size distribution wider and adsorption capacity stronger, resulting in the increase of the impurities adsorption, which was not beneficial to improve the purity of TiO_2_.

Under the fixed slurry concentration of 160 g/L and hydrothermal temperature of 140 °C, with the increasing of hydrothermal time, the purity of TiO_2_ first increased and then decreased, and the results showed that there were interactions between hydrothermal time and the other two factors, as showed in Fig. 1c. Hydrothermal time affected the dissolution, crystallization process, and particle growth for the metatitanic acid. With the extension of hydrothermal time, the TiO^2+^ ions formed by the dissolution of fine metatitanic acid particles would continuously collide and aggregate, forming the colloidal anatase phase crystal nucleus. The new formed crystal core continuously absorbed the TiO^2+^ ions onto its surface, and further deposited and bound, which made the crystal growing continuously, and formed the corresponding primary agglomerates and the secondary aggregates through the hydrolysis, polymerization, condensation and other precipitation-crystallization reactions, which would affect the impurities content absorbed on the surface of the hydrothermal treated metatitanic acid. When the hydrothermal time was short, the dissolution and crystallization process had not been completed, which would lead to adsorb more impurities on the surface of metatitanic acid, resulting in decreasing the purity for the high purity titanium dioxide. On the other hand, when the hydrothermal time was too long, some new precipitates would be formed with smaller particle size, and the aging effect for impurity ions became more significant due to the long time, which led to the enrichment of impurity ions on the metatitanic acid’s surface, resulting in the reduction of the purity. From Fig. 1c, it was obvious that the effects of hydrothermal time on the purity of TiO_2_ were larger than the other two factors.

Based on the results which were generated by the *BBD* method with the responses obtained experimentally for the purity of TiO_2_ (*Y*) (Table 2) the empirical relationship that related the response values and selected factors was obtained as the following equation.

1 $$ Y\, = \,{7}0.{28375}\, + \,0.{166}0{9}X_{1} \, + \,0.{132}0{4}X_{2} \, + \,{2}.{1865}0X_{3} \, + \,{3}.{75} \times {1}0^{{ - {5}}} X_{1} X_{2} {-}{1}.{6875} \times {1}0^{{ - {3}}} X_{1} X_{3} {-}{1}.{8125} \times {1}0^{{{-}{3}}} X_{2} X_{3} {-}{5}.{2} \times {1}0^{{ - {4}}} X_{1}^{2} {-}{4}.{325} \times {1}0^{{ - {4}}} X_{2}^{2} {-}0.{12262}X_{3}^{2} $$

In Eq. (1), the absolute value of each coefficient directly reflected impact of each parameter on the index value, and the positive and negative of the coefficient reflected the direction of its influence. According to Eq. (1), it could be seen that the order of influence of various factors on the purity of TiO_2_ was *X*_3_ > *X*_1_ > *X*_2_, that was, the hydrothermal time > slurry concentration > hydrothermal temperature.

**Figure 2:**
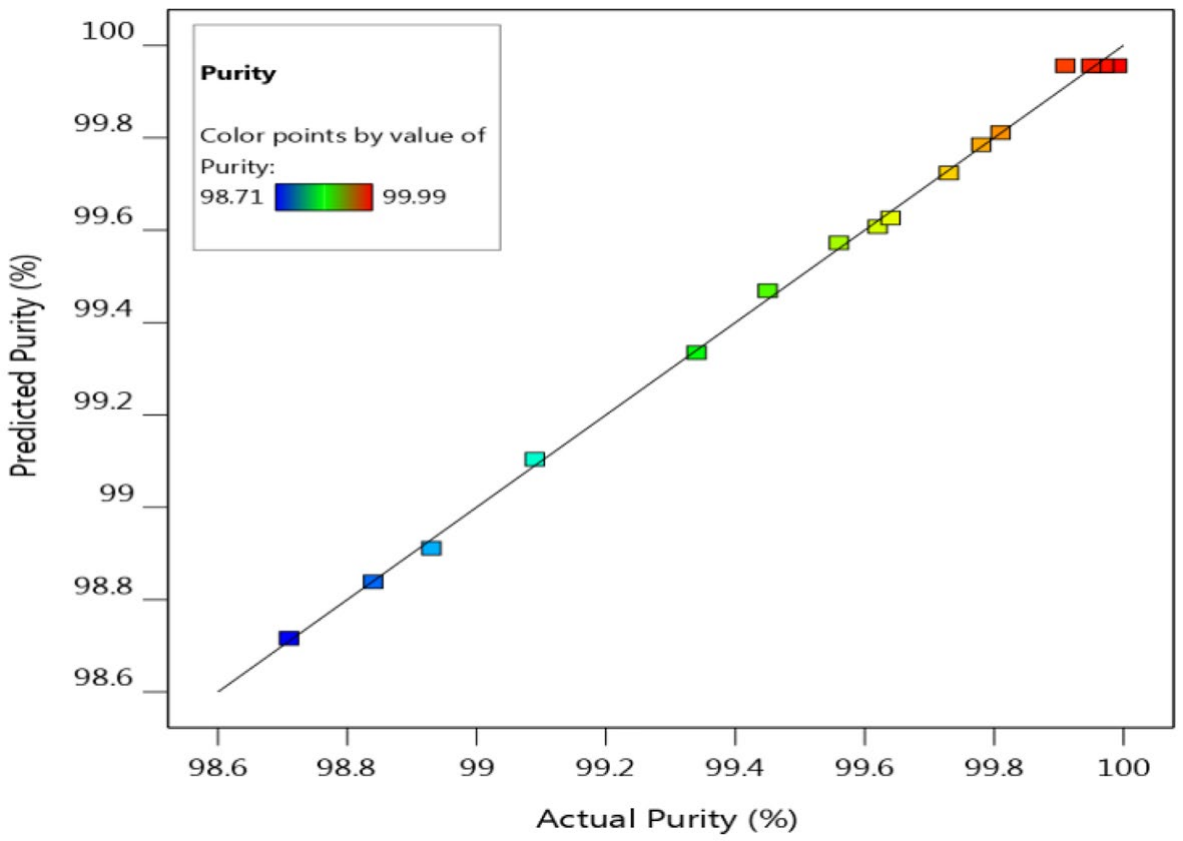
The actual purity values (%) plotted against the predicted values (%) derived from the model of purity of TiO_2_.

The experimental values for the purity of TiO_2_ versus the predicted ones were shown in Fig. 2 and Table 2, and the predicted values were similar to the observed data in all sets of experiments. The predicted purity values agreed well with the experimental values with *R*^2^ = 0.9982 (correlation coefficient) as shown in Table 3, implying that 99.82% of the variations in the TiO_2_ purity, which were illustrated by the selected parameters. The adjusted correlation coefficient (*R*^2^_adj_ = 0.9960) for the purity of TiO_2_ was a corrected goodness-of-fit, and it was also close to the coefficient of determination *R*^2^, which indicated that the regression predicting the purity values approximated very well with the real data.

**Table 3:** Variance analysis of response surface experiments results for the purity of TiO_2_.

Source	Sum of squares	df	Mean square	F value	P-value	Significant
Model	2.848167	9	0.316463	439.0963	< 0.0001	**
*X*_1_	0.086112	1	0.086112	119.4822	< 0.0001	**
*X*_2_	0.117613	1	0.117613	163.1888	< 0.0001	**
*X*_3_	1.17045	1	1.17045	1624.014	< 0.0001	**
*X*_1_*X*_2_	0.0009	1	0.0009	1.248761	0.3007	
*X*_1_*X*_3_	0.018225	1	0.018225	25.28741	0.0015	**
*X*_2_*X*_3_	0.021025	1	0.021025	29.17245	0.001	**
*X*_1_^2^	0.182164	1	0.182164	252.7551	< 0.0001	**
*X*_2_^2^	0.126017	1	0.126017	174.8499	< 0.0001	**
*X*_3_^2^	1.013012	1	1.013012	1405.566	< 0.0001	**
Residual	0.005045	7	0.000721			
Lack of fit	0.001525	3	0.000508	0.577652	0.6599	
Pure error	0.00352	4	0.00088			
Cor total	2.853212	16				
R^2^				0.9982		
**R**^**2**^_**Predj**_ = **0.9895,** **R**^**2**^_**Adj**_ = **0.9960, S/N (signal-to-noise ratio)** = **164.650**						
C.V. %				2.70		

*Indicated that it had a significant impact on the results (P < 0.05), **Indicated that it had a very significant impact on the results.

Variance analysis (Table 3) was obtained to determine the primary and synergistic effects of these conditions that affected the purity of TiO_2_. The model was extremely significant and could be validated by *F* value for *Y* (439.0963), showing that the differences in freezing treatment conditions were highly significant. The *P*-value of this model was much smaller than 0.01, showing that the response surface regression model reached a very significant level. Among them, the primary factors *X*_1_, *X*_2_ and *X*_3_ had a very significant impact on the experimental results (*P* < 0.01). The interaction items *X*_1_*X*_3_ and *X*_2_*X*_3_ had a very significant effect on the experimental results (P < 0.01), while *X*_1_*X*_2_ had no significant impact on the experimental results (P > 0.05). While all the quadratic terms *X*_1_^2^, *X*_2_^2^ and *X*_3_^2^ had a very significant effect on the experimental results (P < 0.01). The correlation coefficient *R*^2^ was close to 1, showing that the correlation between the predicted value of the model and the experimental value was better, which was proved correct as showed in Table 2. Here, the predicted correlation coefficient (*R*^2^_Pred_) was of 0.9895, showing that the model could represent the experimental results well. The value of lack of fit for the model indicated the probability that the predicted value of the model was not match the actual value. The *F*-value of the lack of fit in Table 3 was of 0.6599, larger than 0.100, which indicated that the equation fitted the test results very well. The signal-to-noise ratio was 164.650, which was much larger than 4, and this also showed that this model was reliable. The *C.* *V.* value of the experiments was of 2.70*%*, which was very low, indicating that the operation of the experiments was reliable.

**Figure 3:**
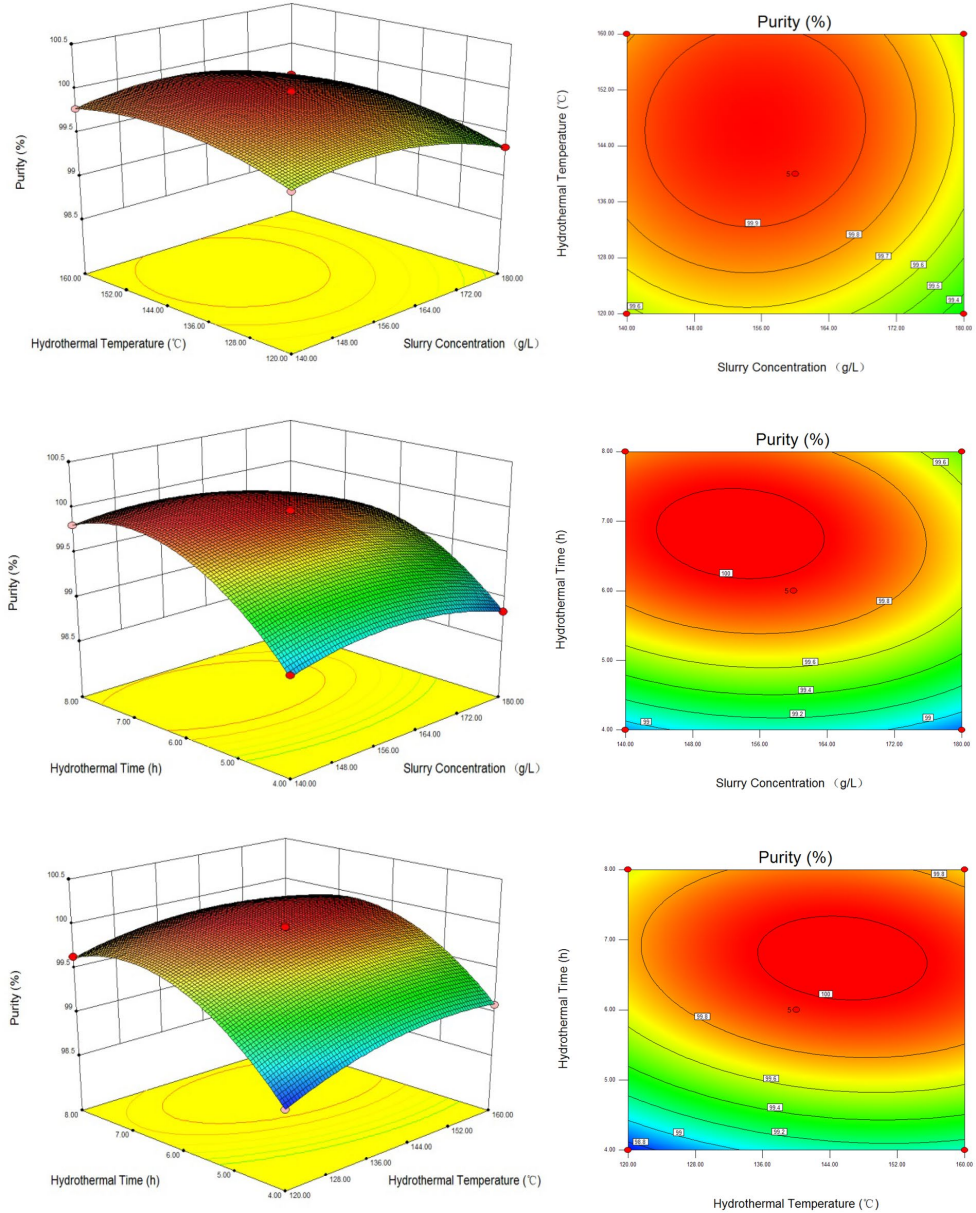
Response surface plots and contour line of effects of interaction between each factor on the purity of TiO_2_.

The 3D diagram and contour map of the response surface in Fig. 3 could intuitively reflect the influence of the interaction of various factors on the purity of TiO_2_ and the value of various factors under optimal conditions. In Fig. 3, as for hydrothermal time and slurry concentration, hydrothermal time and hydrothermal temperature, the response surface curves were steep, the contour lines were closed oval and the response surface was convex, indicating that there was a significant interaction for them, which agreed with the results of variance analysis in in Table 3. The TiO_2_ with high purity could be obtained under suitable hydrothermal conditions, especially for the hydrothermal time and slurry concentration of TiO_2_.

When the hydrothermal time was fixed, the purity of TiO_2_ showed increasing first and then decreasing with the increasing of slurry concentration and hydrothermal temperature, which showed they had synergistic effects. The contour line was a closed incomplete ellipse and the response surface was a weak convex, indicating that the interaction between the slurry concentration and hydrothermal temperature was not significant and had a maximum value, which agreed with the results of variance analysis. When the hydrothermal time was fixed at 6 h, so as to obtain the purity of TiO_2_ above 99.90%, the slurry concentration should be kept in the range of 142.2 ~ 168.4 g/L, as well as the hydrothermal temperature of 132.4 ~ 160.0 °C.

When the hydrothermal temperature was fixed, the purity of TiO_2_ showed increasing first and then decreasing as the slurry concentration and hydrothermal time increasing. The contour line was a closed ellipse and the response surface was a strong convex, indicating that the interaction between the slurry concentration and hydrothermal time was significant and had a maximum value, agreed with the results of variance analysis. When the hydrothermal temperature was of 140 °C, in order to obtain the purity of TiO_2_ more than 99.90*%*, the slurry concentration should be kept in the range of 140.0 ~ 170.9 g/L and the hydrothermal time of 5.71 ~ 7.94 h.

When the slurry concentration was fixed, the purity of TiO_2_ showed also increasing first and then decreasing as the hydrothermal temperature and hydrothermal time increasing. The contour line was a closed ellipse and the response surface was a strong convex, indicating that the interaction between the hydrothermal temperature and hydrothermal time was significant and had a maximum value, consistent with the results of variance analysis. When the slurry concentration was of 160 g/L, in order to obtain the purity above 99.90%, the hydrothermal temperature should be kept in the range of 127.2 ~ 160 °C and the hydrothermal time of 5.63 ~ 7.83 h.

The equation was regressed step by step, and the optimum hydrothermal conditions for the high purity TiO_2_ preparation were determined as the following: slurry concentration 153.95 g/L, hydrothermal temperature 145.12 °C and hydrothermal time 6.78 h. On these optimal hydrothermal conditions, the predicted purity value of TiO_2_ was of 100.062*%*, showing that high purity TiO_2_ with nearly 100*%* could be prepared by controlling the hydrothermal conditions. In order to facilitate the practical operation, the optimal hydrothermal treatment conditions were revised as the following: slurry concentration 154 g/L, hydrothermal temperature 145 °C and hydrothermal time 6.8 h. Under the optimal conditions, the purity of TiO_2_ was of 99.987 ± 0.006%, as showed in Table 4, and the relative deviation was only 0.08%, consistent with the predicted value of the model, indicating that there was a good fit between the predicted value and the experimental actual value, which further verified the reliability of the model.

**Table 4:** Results of verification test.

No	Slurry concentration (g/L)	Hydrothermal temperature (*°*C)	Hydrothermal Time (h)	Purity of TiO_2_ (%)
18	154	145	6.8	99.98
19	154	145	6.8	99.99
20	154	145	6.8	99.99

### Characterization of metatitanic acid and TiO_2_

**Figure 4:**
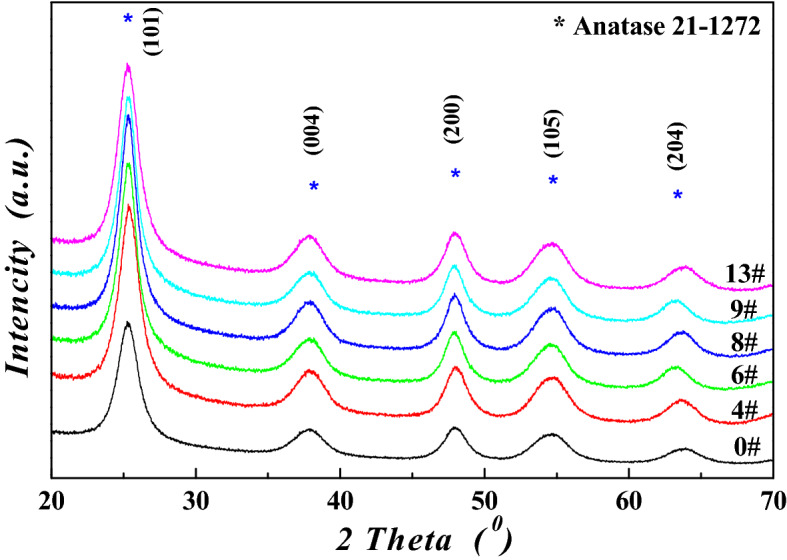
XRD patterns for the metatitanic acid samples.

**Figure 5:**
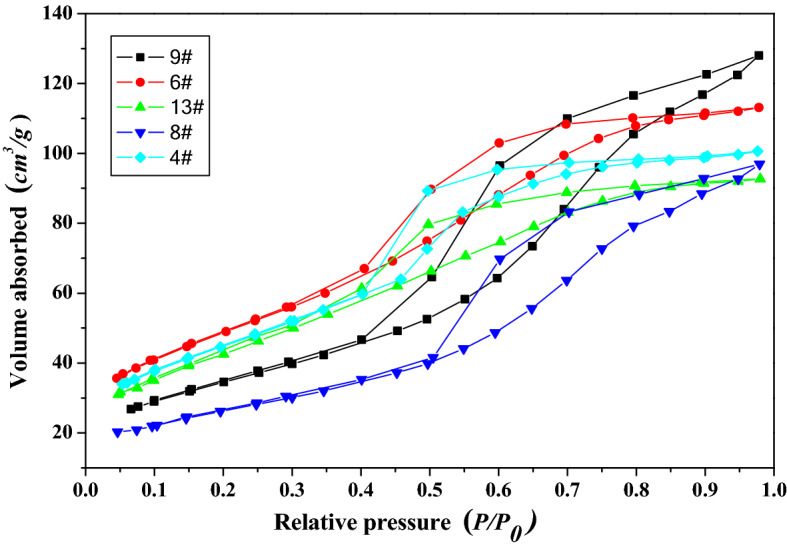
Nitrogen isotherms for the hydrothermal treated metatitanic acid samples.

**Figure 6:**
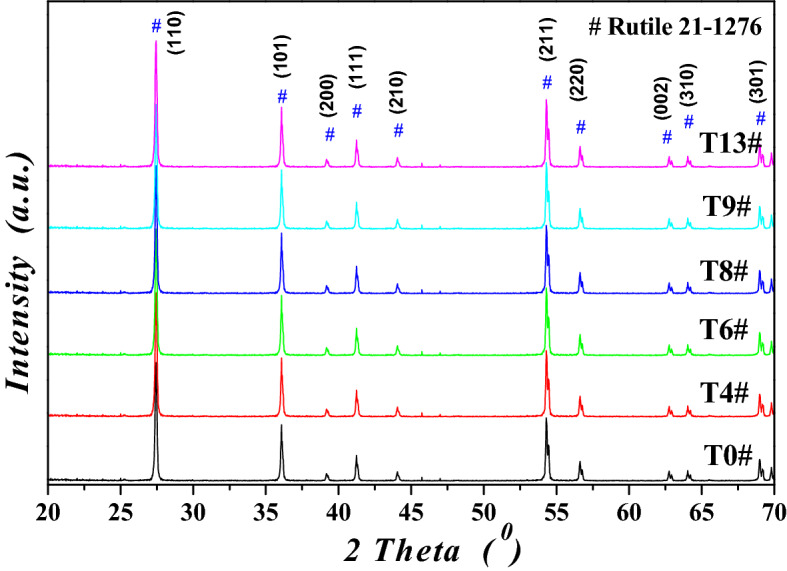
XRD patterns for the high purity TiO_2_ samples.

To investigate the structures and composition of metatitanic acid (abbreviated as *MA*) and TiO_2_ under the different hydrothermal conditions, the samples with experimental numbers of 4#, 6#, 8#, 9# and 13# were selected as the investigation objects, and 0# represented the sample which was without hydrothermal treatment. The XRD patterns for *MA* samples were shown in Fig. 4, and the nitrogen isotherms for the hydrothermal treated *MA* samples were exhibited in Fig. 5, and the XRD patterns for high purity TiO_2_ samples were shown in Fig. 6. The anatase crystal size (*L*_*(101),MA*_) for *MA* samples and the rutile crystal size *L*_*(110)*_ for TiO_2_ samples, the average particle size (*D*_*AV,MA*_), specific surface area (*S*_*BET*_), the TiO_2_ purity for the metatitanic acid and the high purity TiO_2_ were listed in Table 5.

**Table 5:** Effects of hydrothermal conditions on the structure and TiO_2_ content for metatitanic acid and TiO_2_.

Metatitanic acid				High purity TiO_2_			
No	Anatase L _(101),MA_ (nm)	D_AV,MA_ (µm)	S_BET_ (m^2^/g)	No	${\mathrm{D}}_{\mathrm{AV},\mathrm{ Ti}{\mathrm{O}}_{2}}$(µm)	Rutile L_(110)_ (nm)	TiO_2_ Purity (%)
0#	7.3	0.68	227.6	T0#	0.36	92.8	97.86
4#	8.3	0.76	97.5	T4#	0.25	99.7	99.62
6#	8.0	0.82	115.6	T6#	0.30	102	98.84
8#	8.6	0.78	101.9	T8#	0.28	104	99.45
9#	7.8	0.87	127.5	T9#	0.32	108	98.71
13#	8.2	0.71	91.4	T13#	0.23	95.3	99.99

The XRD patterns for all the *MA* samples obviously agreed with the standard spectrum of anatase TiO_2_ phase (*JCPDS* 21-1272) in Fig. 4, indicating they had only anatase phase. All the intensities of diffraction peak for all the metatitanic acid were very small, and the peak shapes were wide and flat, showing that it had a low degree of crystallization. The phase structure of *MA* was mainly amorphous structure, while with a small amount of anatase TiO_2_ crystalline structure, as the ions of the precipitated particles showed the anatase TiO_2_ phase structure in space occupation after bond-valence bonding and deposition growth, and the anatase crystal size for 0# *MA* sample was of 7.3 nm. After hydrothermal treatment, the anatase crystal size increased, range from 7.8 to 8.6 nm, larger than the untreated one. The grain growth would be promoted by increasing the hydrothermal temperature and hydrothermal time, which was helpful to increase crystal size, as showed in Table 5.

The average particle size of the hydrothermal treated *MA* samples (*D*_*AV,MA*_) ranged from 0.87 to 0.71 μm, larger than the untreated *MA* sample with the value of 0.68 μm. The *D*_*AV*_ of sample 13# was the smallest for the hydrothermal treated *MA* with the value of 0.72 μm. The measured *D*_*AV*_ for *MA* corresponded to the secondary aggregation particles, formed by the primary aggregation particles composed of many *MA* crystals. When the crystal size of *MA* was smaller, due to its high surface energy, the primary aggregation particles would be larger, and the final secondary aggregation particles would be smaller. In addition, the size and its distribution for the *MA* crystals and primary aggregation particles would also affect the final secondary aggregation particle size.

As shown in Fig. 5, the isotherms for the hydrothermal treated *MA* samples were the V-type adsorption- desorption isotherm, indicating that the samples had porous structure, which was formed by the accumulation of *MA* particles. The specific surface area (*S*_*BET*_) of the treated *MA* samples varied in the range of 91.4–127.5 m^2^/g, and sample 13# was the smallest, which would reduce the impurity ions adsorption and increase the purity of the prepared titanium dioxide. Comparing with the sample 0# without hydrothermal treatment, the structure of the hydrothermal treated samples had been adjusted, with larger crystal size, more compact structure, weaker colloidal property, resulting in smaller *S*_*BET*_, which could significantly reduce the adsorption of impurities.

The XRD pattern peaks for the calcined TiO_2_ (Fig. 6) were very narrow and sharp, which agreed with the standard rutile TiO_2_ pattern (*JCPDS* 21-1276), showing the calcined TiO_2_ was only with the rutile phase and had high crystallinity, the grain size of TiO_2_ ranged from 95.3 to 108 nm, and sample T13# was with the minimum value of 95.3 nm. During the calcination process, the initial hydrothermal *MA* was with the smaller crystal size and higher activation energy, and it tended to change from the loose anatase phase to the structural compact rutile phase through the ion diffusion of TiO^2+^ on the crystal surface. The appropriate crystal size of *MA* was easier to promote the complete TiO_2_ phase transformation from anatase to rutile, and obtain the more suitable rutile grain size, which was conducive to improve the TiO_2_ purity. The average particle size of the calcined TiO_2_ ranged from 0.23 to 0.32 μm, and sample T13# was with the minimum *D*_*AV*_ value of 0.23 μm. The smaller the *MA* particle size was, the smaller particle size of the obtained high purity TiO_2_ was. Suitable crystal structure and particle size distribution were helpful to increase the purity of TiO_2_, and sample T13# was with the highest purity of 99.99*%*.

**Figure 7:**
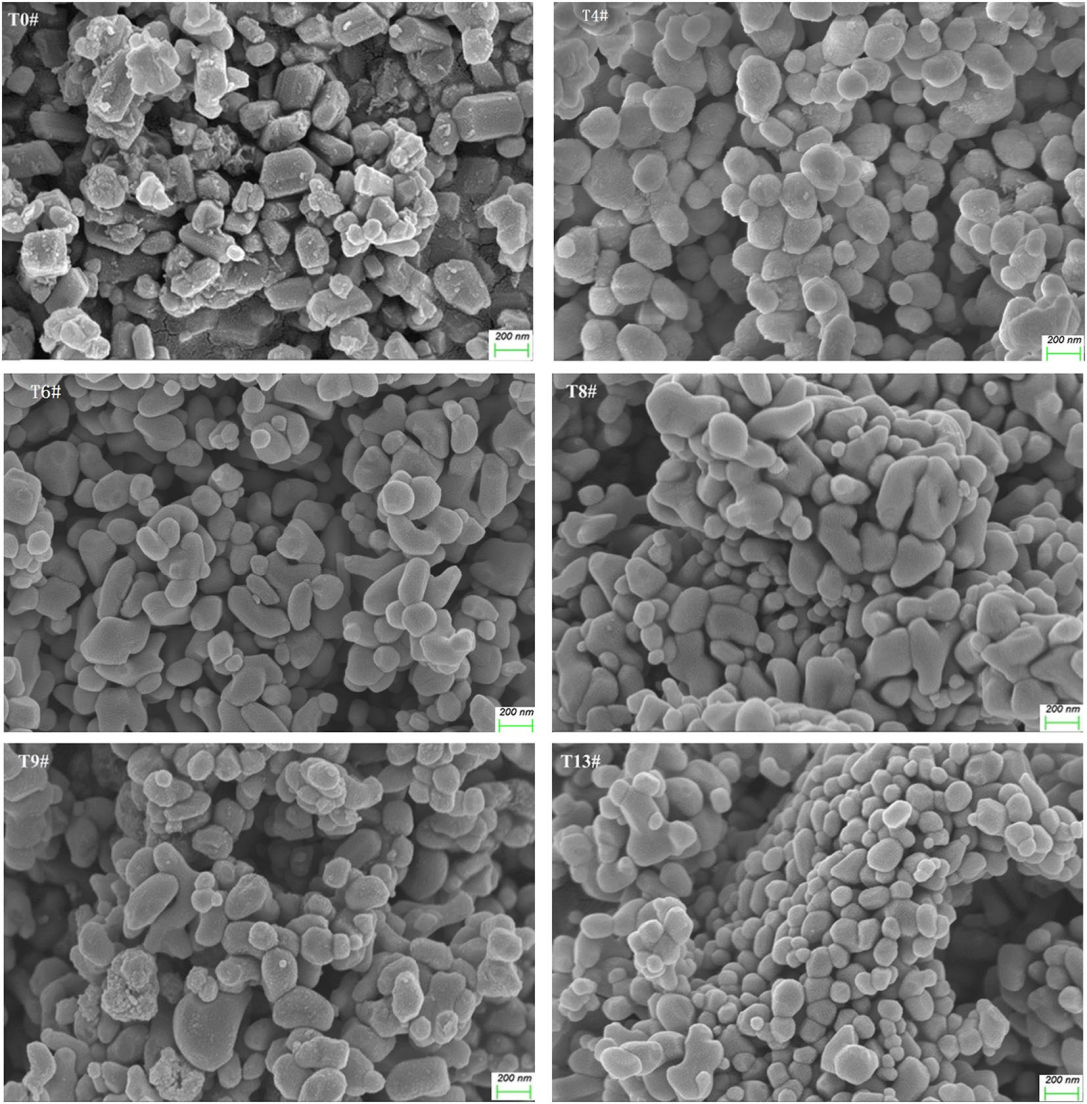
SEM photographs for the high purity TiO_2_ samples.

As shown in Fig. 7, the SEM photographs for TiO_2_ samples showing that the TiO_2_ particles mainly exhibited spherical shape and have obvious contour boundaries, showing they were particles with high crystallinity, in accordance with the XRD analysis. The sample T0# without hydrothermal treatment showed a wide particle size distribution (*PSD*), which agreed with the result that its ${D}_{AV, Ti{O}_{2}}$ was of 0.36 μm. The particle size of the hydrothermal treated samples was mainly distributed ranging from 100 to 350 nm, among them sample T13# was the smallest, mainly distributing in 160–300 nm. Due to its smaller particle size and smaller *S*_*BET*_, the particles for sample T13# were easier to aggregate. The *PSD* of all the other samples was wider than sample T13#, agreeing with the *PSD* analysis results for the high purity TiO_2_. The hydrothermal treatment conditions would affect the dissolution, crystallization rate and growth process for metatitanic acid, and determine the particle size distribution and the *S*_*BET*_ of the hydrothermal treated *MA*, influence the impurities adsorption on the particle surface of *MA*, and finally affected the purity of TiO_2_.

## Conclusions

High purity TiO_2_ was prepared by hydrothermal treatment of metatitanic acid used as the raw material. Based on *BBD* method, the response surface methodology was used to investigate the effects of slurry concentration, hydrothermal temperature and hydrothermal time on the purity of TiO_2_. The hydrothermal treatment conditions had great effects on the structure, particle size and *S*_*BET*_ of the hydrothermal treated metatitanic acid, further affected the impurities adsorption on the metatitanic acid surface, and ultimately affected the purity of TiO_2_. This investigation had proved the applicability by using experimental design to develop the proper response equation and variance analysis, showing that the coefficient of determination of hydrothermal treatment conditions was very high (*R*^2^ = 99.82%). The variables such as the slurry concentration, hydrothermal temperature and hydrothermal time had synergistic effects, and the effect of hydrothermal time on the purity was larger than the other two factors. Furthermore, the experimental values were very close to the predicted ones, the adequacy and validity of the prediction models was examined by using *F* value and *P* value for the purity of TiO_2_, indicating that the response surface regression model reached a very significant level and the operation of the experiments was reliable. At the same time, the verification experiments proved that the predicted data could be achieved at the value of 99.99% under the optimal hydrothermal conditions, further verifying the reliability of the regression model. These results indicated that the optimization by using *RSM* based on the *BBD* approach was an effective means to determine the optimal hydrothermal conditions for preparation of TiO_2_ with high purity.

## Data Availability

All data generated or analyzed during this study are included in this manuscript.

## References

[1] Chen X, Mao SS (2007). Titanium dioxide nanomaterials: Synthesis, properties, modifications, and applications. Chem. Rev..

[2] Liu Y, Tian LH, Tan XY, Li X, Chen XB (2017). Synthesis, properties, and applications of black titanium dioxide nanomaterials. Sci. Bull..

[3] Bai Y, Mora-Sero I, De Angelis F, Bisquert J, Wang P (2014). Titanium dioxide nanomaterials for photovoltaic applications. Chem. Rev..

[4] Bai J, Zhou BX (2014). Titanium dioxide nanomaterials for sensor applications. Chem. Rev..

[5] Ma Y, Wang XL, Jia YS, Chen XB, Han HX, Li C (2014). Titanium dioxide-based nanomaterials for photocatalytic fuel generations. Chem. Rev..

[6] Li W, Elzatahry A, Aldhayan D, Zhao DY (2018). Core-shell structured titanium dioxide nanomaterials for solar energy utilization. Chem. Soc. Rev..

[7] Katir N, Marcotte N, Michlewska S, Ionov M, El Brahmi N, Bousmina M, Majoral JP, Bryszewska M, El Kadib A (2019). Dendrimer for templating the growth of porous catechol-coordinated titanium dioxide frameworks: Toward hemocompatible nanomaterials. ACS Appl. Nano Mater..

[8] Wang MX, Gao Q, Duan H, Ge MQ (2019). Scalable synthesis of high-purity TiO_2_ whiskers via ion exchange method enables versatile applications. RSC Adv..

[9] Li GS, Li LP, Boerio-Goates J, Woodfield BF (2005). High purity anatase TiO_2_ nanocrystals: Near room-temperature synthesis, grain growth kinetics, and surface hydration chemistry. J. Am. Chem. Soc..

[10] O'Regan B, Gratzel M (1991). A low-cost, high-efficiency solar cell based on dye-sensitized colloidal TiO_2_ films. Nature.

[11] Ma TL, Akiyama M, Abe E, Imai I (2005). High-efficiency dye-sensitized solar cell based on a nitrogen-doped nanostructured titania electrode. Nano Lett..

[12] Wang CC, Ying JY (1999). Sol–gel synthesis and hydrothermal processing of anatase and rutile titania nanocrystals. Chem. Mater..

[13] Zhu XF, Zheng SL, Zhang Y, Fang ZGZ, Zhang M, Sun P, Li Q, Zhang Y, Li P, Jin W (2019). Potentially more ecofriendly chemical pathway for production of high-purity TiO_2_ from titanium slag. ACS Sustain. Chem. Eng..

[14] Barringer EA, Bowen HK (1985). High-purity, monodisperse TiO_2_ powders by hydrolysis of titanium tetraethoxide. 1. Synthesis and physical properties. Langmuir.

[15] Akhtar MK, Yun XO, Pratsinis SE (1991). Vapor synthesis of titania powder by titanium tetrachloride oxidation. AIChE J..

[16] Shuang Y, Hou Y, Zhang B, Yang HG (2013). Impurity-free synthesis of cube-like single-crystal anatase TiO_2_ for high performance dye-sensitized solar cell. Ind. Eng. Chem. Res..

[17] Li ZH, Wang ZC, Li G (2016). Preparation of nano-titanium dioxide from ilmenite using sulfuric acid-decomposition by liquid phase method. Powder Technol..

[18] Gao Q, Wang MX, Gao CX, Ge MQ (2021). Light-colored conductive fabric coatings using uniform ATO@TiO_2_ whiskers. J. Mater. Sci..

[19] Gao CX, He SS, Qiu LB, Wang MX, Gao JF, Gao Q (2020). Continuous dry-wet spinning of white, stretchable and conductive fibers of poly(3-hydroxybutyrate-co-4-hydroxybutyrate) and ATO@TiO_2_ nanoparticles for wearable e-textiles. J. Mater. Chem. C.

[20] Tian CX (2022). A novel preparation of high purity TiO_2_ from industrial low concentration TiOSO_4_ solution via short sulfate process. Mater. Sci. Semicon. Proc..

[21] Lu RF, Liu C, Wu JC, Sun W, Sun Q, Dong LC (2021). Process optimization of the extra-adding seeded hydrolysis of TiOSO_4_ to H_2_TiO_3_ by using the unenriched solution for the manufacture of TiO_2_ pigment. J. Cryst. Growth.

[22] Bavykin DV, Parmon VN, Lapkin AA, Walsh FC (2004). The effect of hydrothermal conditions on the mesoporous structure of TiO_2_ nanotubes. J. Mater. Chem..

[23] Yu JG, Wang GH, Cheng B, Zhou MH (2007). Effects of hydrothermal temperature and time on the photocatalytic activity and microstructures of bimodal mesoporous TiO_2_ powders. Appl. Catal. B-Environ..

[24] Suwannaruang T, Kidkhunthod P, Chanlek N, Soontaranon S, Wantala K (2019). High anatase purity of nitrogen-doped TiO_2_ nanorice particles for the photocatalytic treatment activity of pharmaceutical wastewater. Appl. Surf. Sci..

[25] Kighuta K, Gopalan AI, Lee DE, Saianand G, Hou YL, Park SS, Lee KP, Lee JC, Kim WJ (2021). Optimization and modeling of efficient photocatalytic TiO_2_–ZnO composite preparation parameters by response surface methodology. J. Environ. Chem. Eng..

[26] Cho IH, Zoh KD (2007). Photocatalytic degradation of azo dye (Reactive Red 120) in TiO_2_/UV system: Optimization and modeling using a response surface methodology (RSM) based on the central composite design. Dyes Pigments.

[27] Allahveran S, Mehrizad A (2017). Polyaniline/ZnS nanocomposite as a novel photocatalyst for removal of rhodamine 6G from aqueous media: optimization of influential parameters by response surface methodology and kinetic modeling. J. Mol. Liq..

[28] Ferreira SC, Bruns RE, Ferreira HS, Matos GD, David JM, Brandao GC, Dos Santos WNL (2007). Box-Behnken design: An alternative for the optimization of analytical methods. Anal. Chim. Acta.

[29] Tian CX (2016). Calcination intensity on rutile white pigment production via short sulfate process. Dyes Pigments.

[30] Sathyamoorthy S, Moggridge GD, Hounslow MJ (2001). Particle formation during anatase precipitation of seeded titanyl sulfate solution. Cryst. Growth Des..

